# Supplementary material for: Deciphering the transcriptional regulation of the catabolism of lignin-derived aromatics in Rhodococcus opacus PD630
Source: Commun Biol. 2022 Oct 19;5:1109. doi: 10.1038/s42003-022-04069-2 (PMC9582017; doi:10.1038/s42003-022-04069-2)
Supplement: Supplementary file 2 — Supplementary Information [file 42003_2022_4069_MOESM2_ESM.pdf]

Supplementary Information

Deciphering the transcriptional regulation of the catabolism of lignin-derived aromatics in *Rhodococcus opacus* PD630

Jinjin Diao[1], Rhiannon Carr[1], and Tae Seok Moon[1,2,]*

[1] Department of Energy, Environmental and Chemical Engineering, Washington University in St. Louis, St. Louis, Missouri, 63130, USA

[2] Division of Biology and Biomedical Sciences, Washington University in St. Louis, St. Louis, Missouri, 63130, USA

* To whom correspondence should be addressed.
Tae Seok Moon

Supplementary Table 1. Genes of interest in the funneling pathways.

Funneling Pathway	Pathway Genes	Effector Compound	Adjacent TF
Phenol degradation cluster #1	LPD06740 (Flavin reductase, pheB1) and LPD06741 (phenol hydroxylase, PheA1)	Phenol	LPD06739 (*pheR1*)
Phenol degradation cluster #2	LPD06575 (Flavin reductase, pheB2) and LPD06576 (phenol hydroxylase, PheA2)	Phenol	LPD06574 (*pheR2*)
Guaiacol degradation cluster	LPD06578 (Cytochrome P450 CYP255) and LPD06579 (FAD-binding oxidoreductase)	Guaiacol	LPD06577 (*guaR*)
Benzoate degradation cluster	LPD06580 (Benzoate 1,2-dioxygenase subunit alpha, BenA), LPD06581 (Benzoate 1,2-dioxygenase subunit beta, BenB), LPD06582 (Benzoate 1,2-dioxygenase electron transfer component, BenC) and LPD06583	Benzoate	LPD06577 (*guaR*)
4-hydroxybenzoate degradation cluster	LPD06764 (4-hydroxybenzoate hydroxylase, PobA)	4-hydroxybenzoate	LPD06765 (*hbaR1*) and LPD06763 (*hbaR2*)
Vanillate degradation cluster	LPD00563 (Vanillate monooxygenase reductase subunit, VanB), LPD00564 (Hypothetical protein) and LPD00565 (Vanillate O-demethylase oxygenase subunit, VanA)	Vanillate	LPD00561 (*vanR2*) and LPD00562 (*vanR1*)
Catechol branch of β-ketoadipate pathway	LPD06568 (Catechol 1,2-dioxygenase, CatA), LPD06567 (Muconate cycloisomerase, CatB), LPD06566 (Muconate Delta-isomerase, CatC)	Catechol	LPD06569 (*catR*)
Protocatechuic acid branch of the β-ketoadipate pathway	LPD05448 (3-oxoadipate CoA-transferase subunit B, PcaJ), LPD05449 (3-oxoadipate CoA-transferase subunit A, PcaI), LPD05450 (Protocatechuate 3,4-dioxygenase beta chain, PcaH), LPD05451 (Protocatechuate 3,4-dioxygenase alpha chain, PcaG), LPD05452 (3-carboxy-*cis*, *cis*-muconate cycloisomerase, PcaB), LPD05453 (Beta-ketoadipate enol-lactone hydrolase, PcaD), LPD05455 (Acetyl-CoA acyltransferase, PcaF)	Protocatechuic acid	LPD05454 (*pcaR*)

Supplementary Table 2. Plasmids used in this study.

Plasmid	Functional insert	Origin of Replication	Antibiotic Resistance	Length (bp)	Source
pJD016	A selection marker in the center of an ~ 1.2 kb genomic fragment encompassing the target gene LPD06569	p15A (Integrative)	Chloramphenicol	4308	This study, derived from pAGR318
pJD027	A selection marker in the center of an ~ 1.2 kb genomic fragment encompassing the target gene LPD06739	p15A (Integrative)	Chloramphenicol	4308	This study, derived from pAGR318
pJD028	A selection marker in the center of an ~ 1.2 kb genomic fragment encompassing the target gene LPD06574	p15A (Integrative)	Chloramphenicol	4308	This study, derived from pAGR318
pJD029	A selection marker in the center of an ~ 1.2 kb genomic fragment encompassing the target gene LPD06577	p15A (Integrative)	Chloramphenicol	4308	This study, derived from pAGR318
pJD030	A selection marker in the center of an ~ 1.2 kb genomic fragment encompassing the target gene LPD06763	p15A (Integrative)	Chloramphenicol	4308	This study, derived from pAGR318
pJD031	A selection marker in the center of an ~ 1.2 kb genomic fragment encompassing the target gene LPD06765	p15A (Integrative)	Chloramphenicol	4308	This study, derived from pAGR318
pJD032	A selection marker in the center of an ~ 1.2 kb genomic fragment encompassing the target gene LPD00561	p15A (Integrative)	Chloramphenicol	4343	This study, derived from pAGR318
pJD033	A selection marker in the center of an ~ 1.2 kb genomic fragment encompassing the target gene LPD00562	p15A (Integrative)	Chloramphenicol	4343	This study, derived from pAGR318
pJD034	A selection marker in the center of an ~ 1.2 kb genomic fragment encompassing the target gene LPD06699	p15A (Integrative)	Chloramphenicol	4308	This study, derived from pAGR318
pJD035	pConstitutive.Che9c60.Che9c61	pB264	Kanamycin	6270	This study derived from pDD82
pJD049	A selection marker in the center of an ~ 1.2 kb genomic fragment encompassing the target gene LPD06764	p15A (Integrative)	Chloramphenicol	4308	This study, derived from pAGR318
pJD050	A selection marker in the center of an ~ 1.2 kb genomic fragment encompassing the target gene LPD06739	p15A (Integrative)	Gentamycin	4580	This study, derived from pAGR318
pJD056	A selection marker in the center of an ~ 1.2 kb genomic fragment	p15A (Integrative)	Chloramphenicol	4308	This study, derived from pAGR318

	encompassing the target gene LPD06740				
pJD057	A selection marker in the center of an ~ 1.2 kb genomic fragment encompassing the target gene LPD06575	p15A (Integrative)	Chloramphenicol	4308	This study, derived from pAGR318
pJD070	pLPD06580.GFP+	pAL5000/pMB1	Hygromycin B	4887	This study, derived from pDD302
pJD071	pLPD05450.GFP+	pAL5000/pMB1	Kanamycin	4815	This study, derived from pDD302
pJD074	pLPD05449.GFP+	pAL5000/pMB1	Kanamycin	4815	This study, derived from pDD302
pJD075	A selection marker in the center of an ~ 1.2 kb genomic fragment encompassing the target gene LPD05454	p15A (Integrative)	Chloramphenicol	4387	This study, derived from pAGR318
pDD281	pBAD.dCas9$_{sth}$.pT7.sgRNA PHE_1	pAL5000	Gentamycin	9205	This study, derived from pDD247

Supplementary Table 3. Strains used in this study. N/A, not applicable.

Strain name	Genus	Species	Strain	Plasmid Contained
sJD_R_000	*Rhodococcus*	*opacus*	WT	N/A
sJD_R_003	*Rhodococcus*	*opacus*	ΔLPD06569::CmR	N/A
sJD_R_004	*Rhodococcus*	*opacus*	ΔLPD06569::CmR	pRH033
sJD_R_006	*Rhodococcus*	*opacus*	ΔLPD00562::CmR	N/A
sJD_R_007	*Rhodococcus*	*opacus*	WT	pDD301
sJD_R_008	*Rhodococcus*	*opacus*	ΔLPD06739::CmR	N/A
sJD_R_009	*Rhodococcus*	*opacus*	ΔLPD06763::CmR	N/A
sJD_R_010	*Rhodococcus*	*opacus*	ΔLPD06739::CmR	pRH033
sJD_R_011	*Rhodococcus*	*opacus*	ΔLPD00561::CmR	N/A
sJD_R_012	*Rhodococcus*	*opacus*	ΔLPD06698::CmR	N/A
sJD_R_013	*Rhodococcus*	*opacus*	ΔLPD06765::CmR	N/A
sJD_R_014	*Rhodococcus*	*opacus*	ΔLPD06577::CmR	N/A
sJD_R_015	*Rhodococcus*	*opacus*	ΔLPD06765::CmR	pDD300
sJD_R_016	*Rhodococcus*	*opacus*	ΔLPD06577::CmR	pDD302
sJD_R_017	*Rhodococcus*	*opacus*	ΔLPD06577::CmR	pDD303
sJD_R_018	*Rhodococcus*	*opacus*	ΔLPD06698::CmR	pRH037
sJD_R_019	*Rhodococcus*	*opacus*	ΔLPD06574::CmR	N/A
sJD_R_020	*Rhodococcus*	*opacus*	ΔLPD06764::CmR	N/A
sJD_R_021	*Rhodococcus*	*opacus*	ΔLPD06765::CmR	pDD303
sJD_R_022	*Rhodococcus*	*opacus*	ΔLPD06574::CmR	pRH036
sJD_R_024	*Rhodococcus*	*opacus*	ΔLPD06765::CmR	pRH033
sJD_R_025	*Rhodococcus*	*opacus*	WT	pJD035
sJD_R_028	*Rhodococcus*	*opacus*	WT	pRH037
sJD_R_030	*Rhodococcus*	*opacus*	ΔLPD06574::CmR	pRH033
sJD_R_031	*Rhodococcus*	*opacus*	ΔLPD00561::CmR	pDD301
sJD_R_032	*Rhodococcus*	*opacus*	ΔLPD00562::CmR	pDD301
sJD_R_033	*Rhodococcus*	*opacus*	WT	pRH036
sJD_R_034	*Rhodococcus*	*opacus*	ΔLPD06739::CmR	pRH036
sJD_R_035	*Rhodococcus*	*opacus*	ΔLPD06574::CmR ΔLPD06739::GentR	N/A
sJD_R_036	*Rhodococcus*	*opacus*	WT	pDD054
sJD_R_037	*Rhodococcus*	*opacus*	ΔLPD06569::CmR	pDD054
sJD_R_038	*Rhodococcus*	*opacus*	ΔLPD06764::CmR	pDD303
sJD_R_039	*Rhodococcus*	*opacus*	ΔLPD06765::CmR	pDD303
sJD_R_040	*Rhodococcus*	*opacus*	ΔLPD06740::CmR	N/A
sJD_R_044	*Rhodococcus*	*opacus*	ΔLPD06574::CmR ΔLPD06739::GentR	pRH033
sJD_R_045	*Rhodococcus*	*opacus*	ΔLPD06569::CmR	pRH036
sJD_R_046	*Rhodococcus*	*opacus*	ΔLPD06569::CmR	pDD302
sJD_R_047	*Rhodococcus*	*opacus*	ΔLPD06569::CmR	pDD303
sJD_R_048	*Rhodococcus*	*opacus*	ΔLPD06574::CmR	pDD302
sJD_R_049	*Rhodococcus*	*opacus*	ΔLPD06739::CmR	pDD302
sJD_R_050	*Rhodococcus*	*opacus*	ΔLPD06574::CmR ΔLPD06739::GentR	pDD302

sJD_R_053	*Rhodococcus*	*opacus*	WT	pDD300
sJD_R_055	*Rhodococcus*	*opacus*	p6575.T7RNAP::HygR_NIII	pDD267
sJD_R_056	*Rhodococcus*	*opacus*	p6575.T7RNAP::HygR_NIII	pDD281
sJD_R_059	*Rhodococcus*	*opacus*	WT	pJD071
sJD_R_060	*Rhodococcus*	*opacus*	ΔLPD06574::CmR<br>ΔLPD06739::GentR<br>ΔLPD06577::SpecR	N/A
sJD_R_061	*Rhodococcus*	*opacus*	ΔLPD06574::CmR<br>ΔLPD06739::GentR<br>ΔLPD06577::SpecR	pJD070
sJD_R_063	*Rhodococcus*	*opacus*	ΔLPD05454::CmR	N/A
sJD_R_065	*Rhodococcus*	*opacus*	ΔLPD05454::CmR	pJD071
sJD_R_066	*Rhodococcus*	*opacus*	WT	pJD074
sJD_R_067	*Rhodococcus*	*opacus*	ΔLPD05454::CmR	pJD074

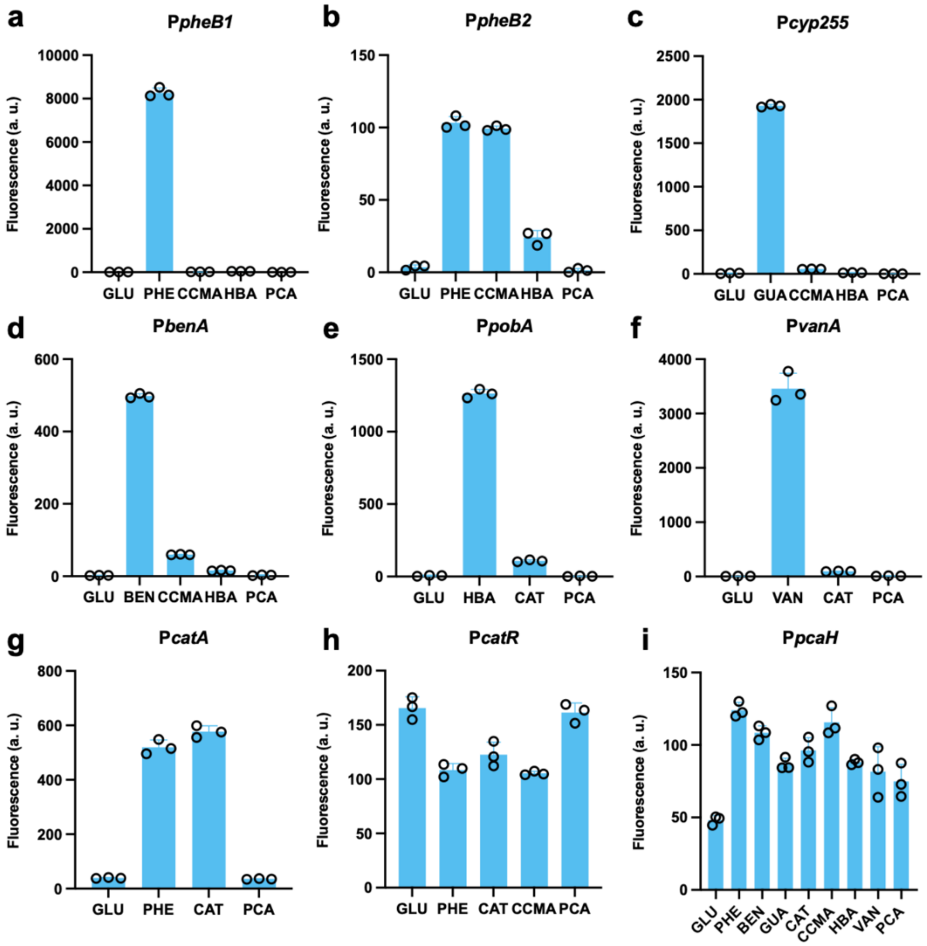

Supplementary Figure 1. Selective responses of the aromatic responsive promoters. The fluorescence response of each construct in the WT strain was tested in the presence of 1 g/L glucose (GLU) or a combination of 1 g/L GLU and 0.3 g/L of one of the following: phenol (PHE), benzoate (BEN), guaiacol (GUA), catechol (CAT), 4-hydroxybenzoate (HBA), vanillate (VAN), protocatechuic acid (PCA), or 5 g/L *cis*-*cis* muconate (CCMA). All fluorescence values were measured in the early stationary phase and normalized to OD_{600}. All values represent the mean of triplicate cultures, with error bars depicting the standard deviation from that mean.

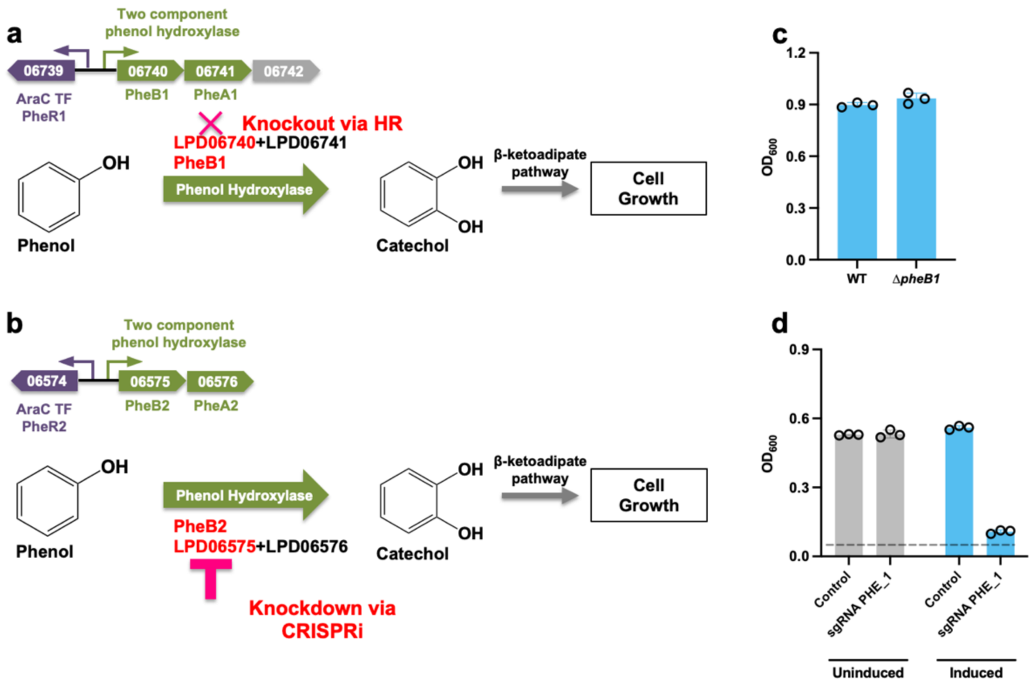

Supplementary Figure 2. Functional analysis of two potential phenol funneling pathways. a, **b**, The *R. opacus* PD630 genome includes two annotated phenol degradation clusters, which are illustrated with their corresponding pathways. For pathway **a**, the mutant Δ*pheB1* (ΔLPD06740) was generated via homologous recombination; for pathway **b**, complete deletion of *pheB2* (LPD06575) from the genome could not be achieved, so CRISPRi was applied to downregulate its expression level. **c**, The cell growths (OD_{600}) of the WT and the Δ*pheB1* strain were comparable when 0.7 g/L phenol was provided as the sole carbon source. **d**, a T7 RNAP-based CRISPRi system was used to target the phenol hydroxylase gene *pheB2* (LPD06575). In this system, T7 RNAP expression is regulated by the phenol-inducible promoter, sgRNA expression is controlled by the T7 promoter, and expression of a codon-optimized version of *dcas9* ($dcas9_{Sth1}$) is arabinose-inducible via the pBAD promoter. After 60 hours, the cell culture density (OD_{600}; dashed line represents the initial OD_{600} = 0.05) was measured for the control strain ($dcas9_{Sth1}$ and T7 RNAP, but no sgRNA) and a strain expressing both $dcas9_{Sth1}$ and sgRNA PHE_1. Induction of the CRISPRi circuit resulted in a substantial growth defect. All values represent the mean of triplicate cultures, with error bars depicting the standard deviation from that mean.

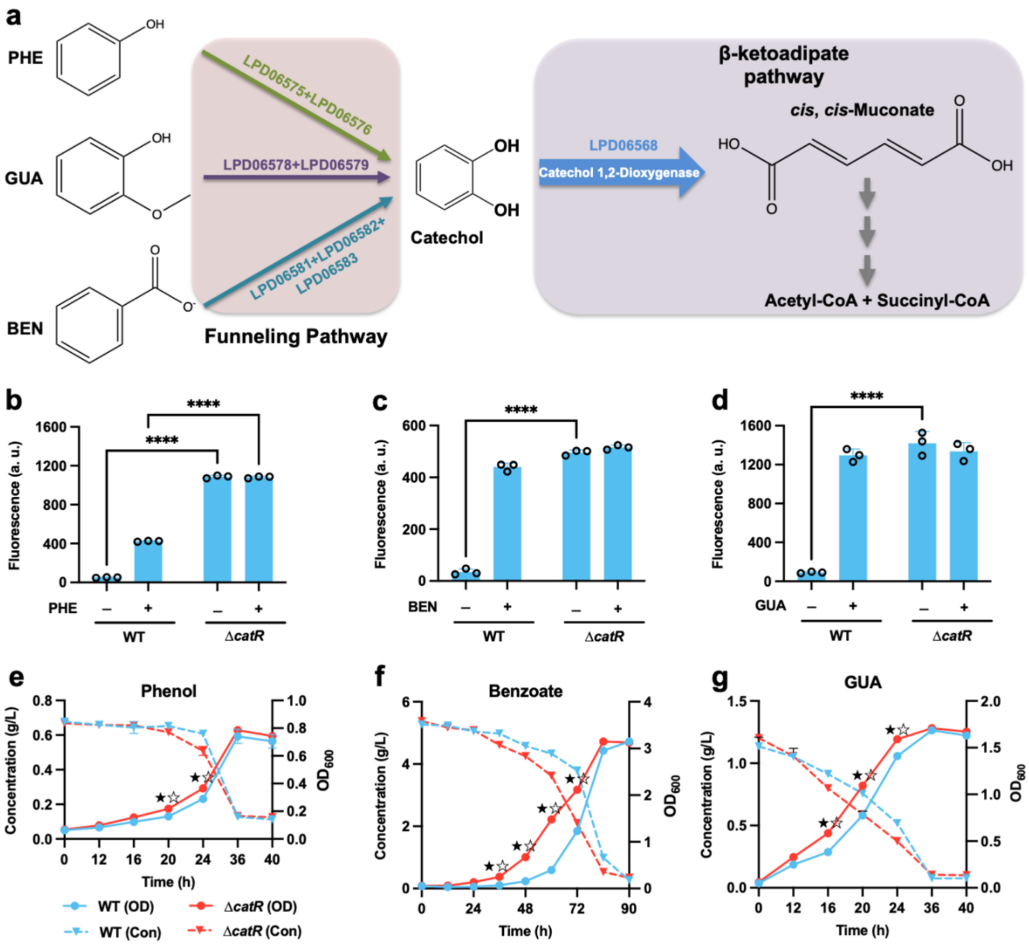

Supplementary Figure 3. Functional analysis of the transcription factor *catR* (LPD06569). a, Schematic diagram of the degradation of phenol (PHE), guaiacol (GUA), and benzoate (BEN) by *R. opacus* PD630. All three compounds are converted via compound-specific funneling pathways to catechol, which feeds into the β-ketoadipate pathway and is converted into acetyl-CoA and succinyl-CoA to support cell growth. **b**-**d**, Analysis of the function of *catR*. The construct P*catA*-GFP+ was expressed in both the WT and the transcription factor deletion mutant strains. The normalized fluorescence of the WT and Δ*catR* strains in response to phenol (**b**), benzoate (**c**), and guaiacol (**d**) was measured in the early stationary phase and normalized to OD_{600} (****, P <0.0001, unpaired two-tailed t-test). All cultures contained 1 g/L glucose with (+) or without (**-**) the respective compounds (PHE 0.3 g/L, BEN 1 g/L, GUA 0.3 g/L). **e**-**g**, Comparisons of cell growth (OD) and aromatics consumption (Con) between the WT and Δ*catR* strains when fed with different aromatic monomers: 0.7 g/L phenol (**e**), 5 g/L benzoate (**f**), or 1.25 g/ L guaiacol (**g**). The variations in the changes of cell density and aromatic concentration of the mutant were compared against those of the WT control (★, P <0.05 for OD; ☆, P <0.05 for Con; two-tailed mixed model ANOVA with Sidak's multiple comparisons). All values represent the mean of triplicate cultures, with error bars depicting the standard deviation from that mean. Gene codes correspond to NCBI reference sequence CP003949.1.

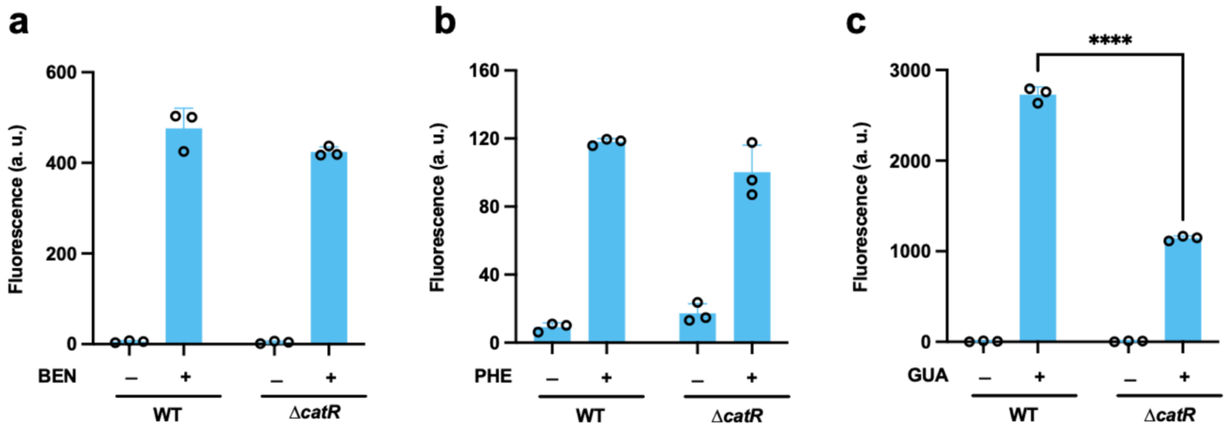

Supplementary Figure 4. Aromatic compound-dependent response of three promoters in the Δ*catR* mutant. The upstream promoter regions of *benA* (LPD06580), *pheB2* (LPD06575), and cytochrome P450 *CYP255* (LPD06578) were cloned ahead of *gfp+*, and each resulting fusion construct was expressed in both WT and Δ*catR* strains. **a**, The normalized fluorescence of P*benA*-GPF+ in the WT and Δ*catR* strains grown with and without supplemental benzoate (BEN). **b**, The normalized fluorescence of P*pheB2*-GPF+ in the WT and Δ*catR* strains grown with and without supplemental phenol (PHE). **c**, The normalized fluorescence of P*cyp255*-GPF+ in the WT and Δ*catR* strains grown with and without supplemental guaiacol (GUA). Guaiacol-induced fluorescence in the Δ*catR* background was significantly reduced from that of the WT (****, *P* <0.0001, unpaired two-tailed *t*-test). For the fluorescence assays, all cultures contained 1 g/L glucose with (+) or without (-) the noted aromatic compound (1.0 g/L BEN, 0.3 g/L PHE, or 0.3 g/L GUA). All fluorescence values were measured in the early stationary phase and normalized to OD_{600}. All values represent the mean of triplicate cultures, with error bars depicting the standard deviation from that mean.

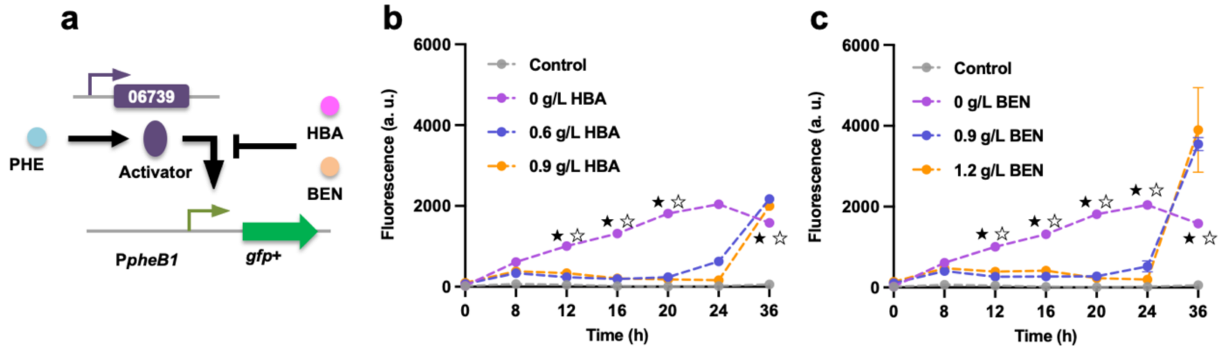

Supplementary Figure 5. Promoter activity of P*pheB1* in response to phenol and either 4-hydroxybenzoate or benzoate. a, The proposed working model for the promoter P*pheB1* during growth on minimal medium with phenol (PHE) and either 4-hydroxybenzoate (HBA) or benzoate (BEN). Promoters are represented as small arrows, and genes are shown with LPD gene numbers from the NCBI database (Refseq, CP003949.1). **b**, The normalized fluorescence was measured in response to varying the ratio of aromatic compounds, with constant 0.3 g/L PHE and variable HBA (0, 0.6, or 0.9 g/L). **c**, The normalized fluorescence was measured in response to varying the ratio of aromatic compounds, with constant 0.3 g/L PHE and variable BEN (0, 0.9, or 1.2 g/L). In addition to aromatics, all cultures were fed with 1 g/L glucose; fluorescence values were measured and normalized to OD_{600}. Fluorescence of P*pheB1*-GFP+ was observed to decrease in the presence of HBA or BEN (★: $P<0.05$ for 0.6 g/L HBA or 0.9 g/L BEN, ☆: $P<0.05$ for 0.9 g/L HBA or 1.2 g/L BEN; two-tailed mixed model ANOVA with Sidak's multiple comparisons). All values represent the mean of triplicate cultures, with error bars depicting the standard deviation from that mean.

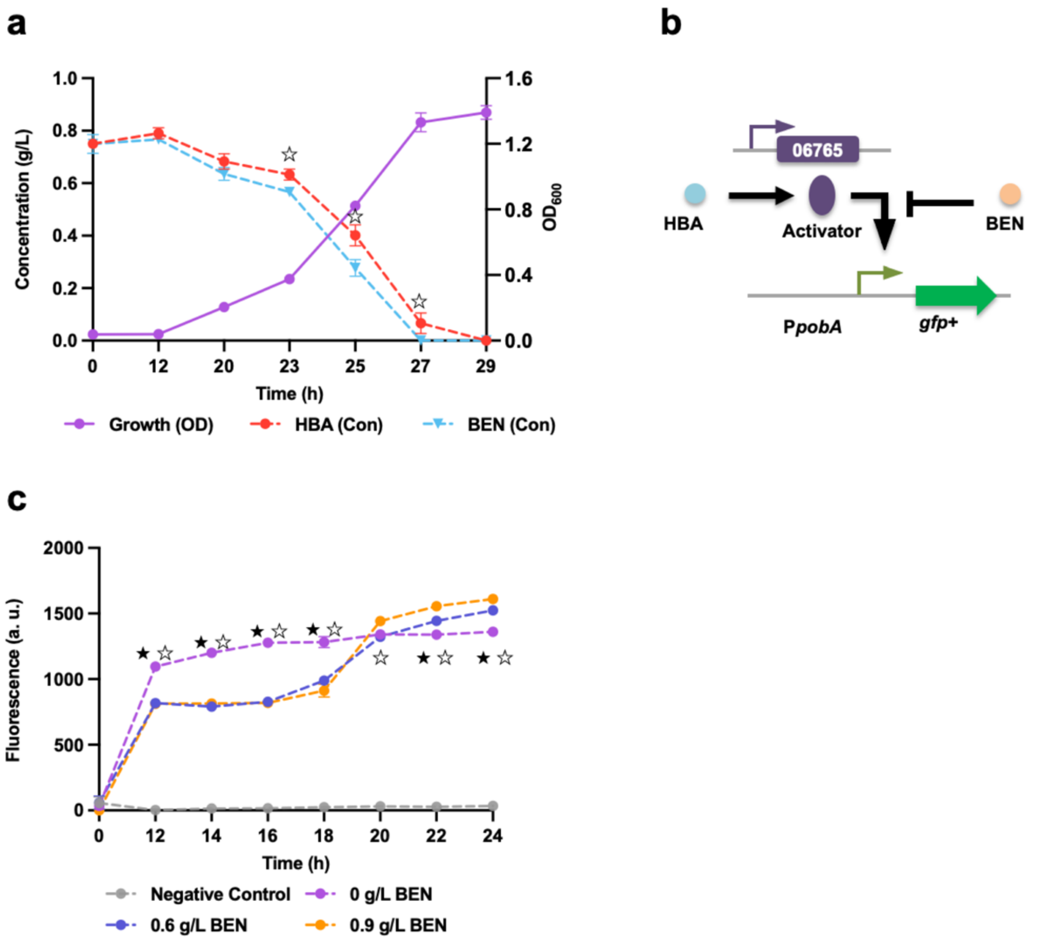

Supplementary Figure 6. Preferential utilization of benzoate over 4-hydroxybenzoate by *R. opacus*. a, Utilization profiles of WT cell culture in minimal medium co-fed with 0.75 g/L benzoate (BEN) and 0.75 g/L 4-hydroxybenzoate (HBA) as carbon sources. At each time point, 200 µL of the cell suspension was removed to measure the cell density (OD), and then spun down to extract the supernatant for HPLC measurement of aromatic concentrations (Con). A significant delay in consumption of HBA was observed relative to that of BEN (☆, $P < 0.05$, two-tailed mixed model ANOVA with Sidak's multiple comparisons). **b**, The proposed working model for the promoter P*pobA* during growth on minimal medium with HBA and BEN. Promoters are represented as small arrows, and genes are shown with LPD gene numbers from the NCBI database (Refseq, CP003949.1). **c**, The normalized fluorescence was measured for different ratios of aromatic compounds, with constant 0.5 g/L HBA and varied BEN (0, 0.6, or 0.9 g/L). In addition to BEN and HBA, all cultures were fed with 1 g/L glucose; fluorescence values were measured and normalized to OD_{600}. The fluorescence of P*pobA*-GFP+ demonstrated an obvious delay and ultimately stronger fluorescence when cells were provided with BEN (★: $P < 0.05$ for 0.6 g/L BEN, ☆: $P < 0.05$ for 0.9 g/L BEN; two-tailed mixed model ANOVA with Sidak's multiple comparisons). All values represent the mean of triplicate cultures, with error bars depicting the standard deviation from that mean.

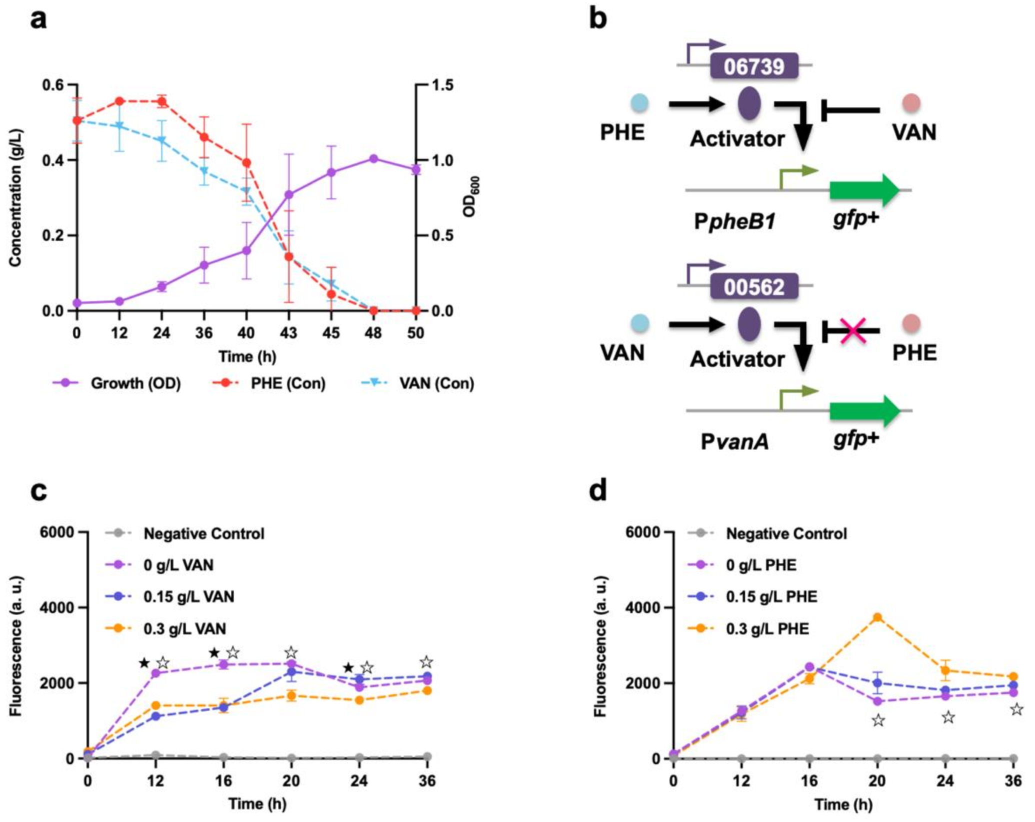

Supplementary Figure 7. Utilization of phenol and vanillate in *R. opacus* is concurrent but shows asymmetric effects on funneling pathways. **a**, Utilization profiles of WT cell culture in minimal medium co-fed with 0.5 g/L phenol (PHE) and 0.5 g/L vanillate (VAN) as carbon sources. At each time point, 200 μL of the cell suspension was removed to measure cell density (OD), and then spun down to extract the supernatant for HPLC measurement of aromatic concentrations (Con). No statistical difference was found in the change in Con for either compound. **b**, The proposed working model for the promoters P*pheB1* (upper) and P*vanA* (lower) during growth in minimal medium with PHE and VAN. Promoters are represented as small arrows, and genes are shown with LPD gene numbers from the NCBI database (Refseq, CP003949.1). **c**, The normalized fluorescence was measured in response to the varying ratio of aromatic compounds, with constant 0.3 g/L PHE and variable VAN (0, 0.15, or 0.3 g/L). **d**, The normalized fluorescence was measured in response to the varying ratio of aromatic compounds, with constant 0.3 g/L VAN and variable PHE (0, 0.15 or 0.3 g/L). In addition to PHE and VAN, all cultures (**c** and **d**) were fed with 1 g/L glucose; fluorescence values were measured and normalized to OD_{600}. The fluorescence of P*pheB1*-GFP+ (**c**) decreased in the presence of VAN, but the fluorescence of P*vanA*-GFP+ (**d**) increased when cells were provided with 0.3 g/L PHE (★: P <0.05 for 0.15 g/L PHE or VAN, ☆: P <0.05 for 0.3 g/L PHE or VAN; two-tailed mixed model ANOVA with Sidak's multiple comparisons). All values represent the mean of triplicate cultures, with error bars depicting the standard deviation from that mean.

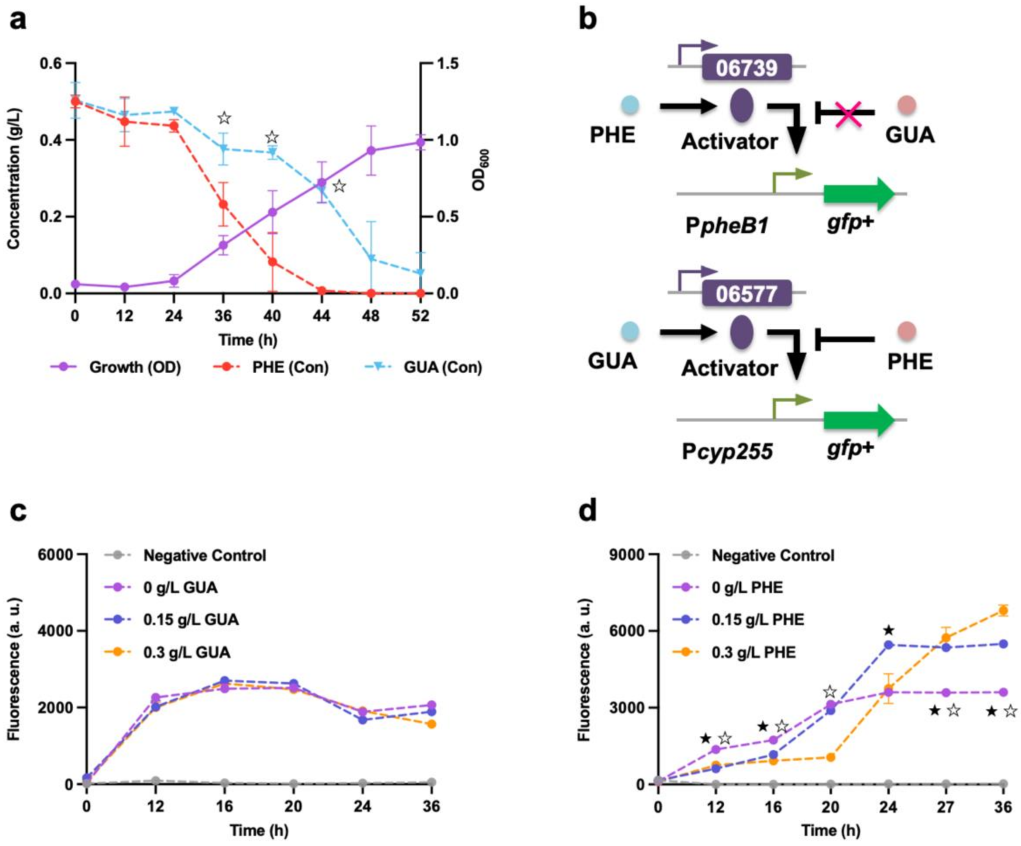

Supplementary Figure 8. Preferential utilization of phenol over guaiacol by *R. opacus*. a, Utilization profiles of WT cell culture in minimal medium co-fed with 0.5 g/L phenol (PHE) and 0.5 g/L guaiacol (GUA) as carbon sources. At each time point, 200 μL of the cell suspension was removed to measure the cell density (OD), and then spun down to extract the supernatant for HPLC measurement of aromatic concentrations (Con). Consumption of GUA was significantly delayed versus that of PHE (☆, $P < 0.05$, two-tailed mixed model ANOVA with Sidak's multiple comparisons). **b**, The proposed working model for the promoters P*pheB1* (upper) and P*cyp255* (lower) during growth in minimal medium with PHE and GUA; promoters are represented as small arrows and genes are shown with LPD gene numbers from the NCBI database (Refseq, CP003949.1). **c**, The normalized fluorescence was measured in response to varied ratios of aromatic compounds, with constant 0.3 g/L PHE and variable GUA (0, 0.15, or 0.3 g/L). **d**, The normalized fluorescence was measured in response to varied ratios of aromatic compounds, with constant 0.3 g/L GUA and variable PHE (0, 0.15, or 0.3 g/L). In addition to PHE and GUA, all cultures (**c** and **d**) were fed with 1 g/L glucose; fluorescence values were measured and normalized to OD_{600}. Fluorescence of P*pheB1*-GFP+ (**c**) was unaffected by the presence of GUA, but P*cyp255*-GFP+ (**d**) demonstrated delayed but ultimately stronger fluorescence when cells were provided with PHE (★: $P < 0.05$ for 0.15 g/L PHE or GUA, ☆: $P < 0.05$ for 0.3 g/L PHE or GUA; two-tailed mixed model ANOVA with Sidak's multiple comparisons). All values represent the mean of triplicate cultures, with error bars depicting the standard deviation from that mean.

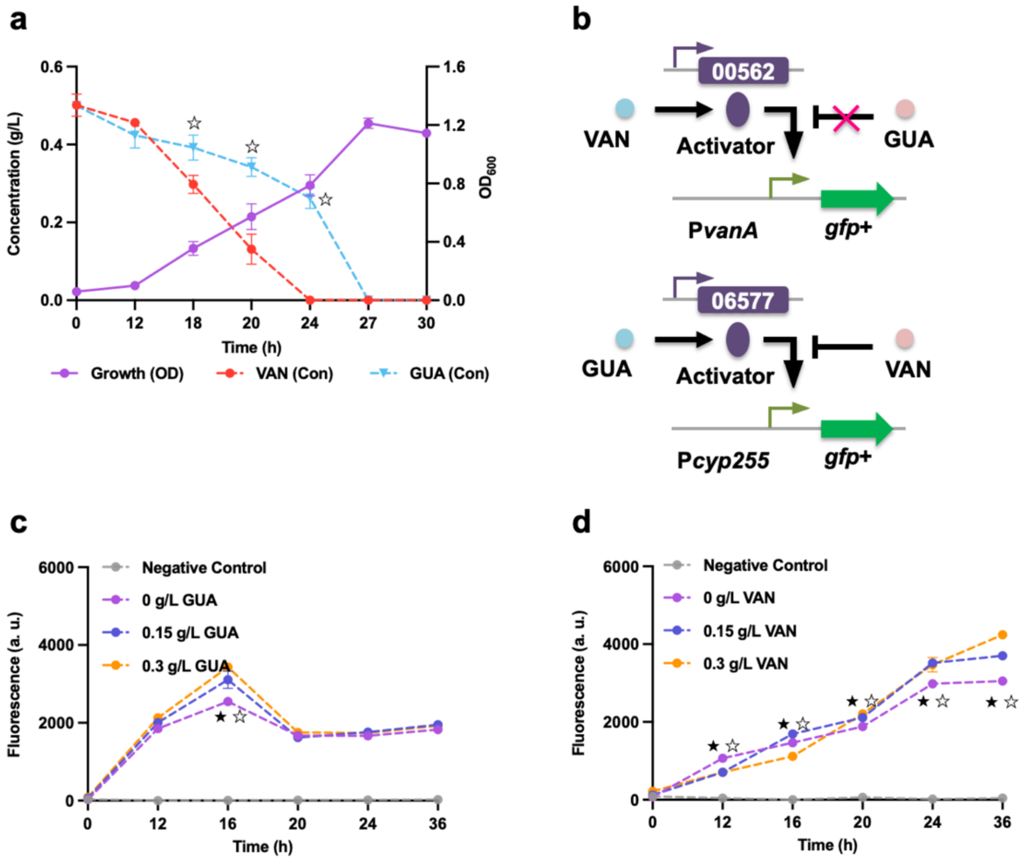

Supplementary Figure 9. Preferential utilization of vanillate over guaiacol in *R. opacus*. a, Utilization profiles of WT cell culture in minimal medium co-fed with 0.5 g/L vanillate (VAN) and 0.5 g/L guaiacol (GUA) as carbon sources. At each time point, 200 µL of the cell suspension was removed to measure cell density (OD), and then spun down to extract the supernatant for HPLC measurement of aromatic concentrations (Con). A significant delay in consumption of GUA was observed versus that of VAN (☆, $P < 0.05$, two-tailed mixed model ANOVA with Sidak's multiple comparisons). **b**, The proposed working model for the promoters P*vanA* (upper) and P*cyp255* (lower) during growth in minimal medium with VAN and GUA; promoters are represented as small arrows and genes are shown with LPD gene numbers from the NCBI database (Refseq, CP003949.1). **c**, The normalized fluorescence was measured in response to varied ratios of aromatic compounds, with constant 0.3 g/L VAN and variable GUA (0, 0.15, or 0.3 g/L). **d**, The normalized fluorescence was measured in response to varied ratios of aromatic compounds, with constant 0.3 g/L GUA and variable VAN (0, 0.15, or 0.3 g/L). In addition to VAN and GUA, all cultures (**c** and **d**) were fed with 1 g/L glucose; fluorescence values were measured and normalized to OD_{600}. The fluorescence of P*vanA*-GFP+ (**c**) was largely unaffected by the presence of GUA, but the fluorescence of P*cyp255*-GFP+ (**d**) demonstrated a delay and ultimately stronger fluorescence when cells were provided with VAN (★: $P < 0.05$ for 0.15 g/L VAN or GUA, ☆: $P < 0.05$ for 0.3 g/L VAN or GUA; two-tailed mixed model ANOVA with Sidak's multiple comparisons). All values represent the mean of triplicate cultures, with error bars depicting the standard deviation from that mean.

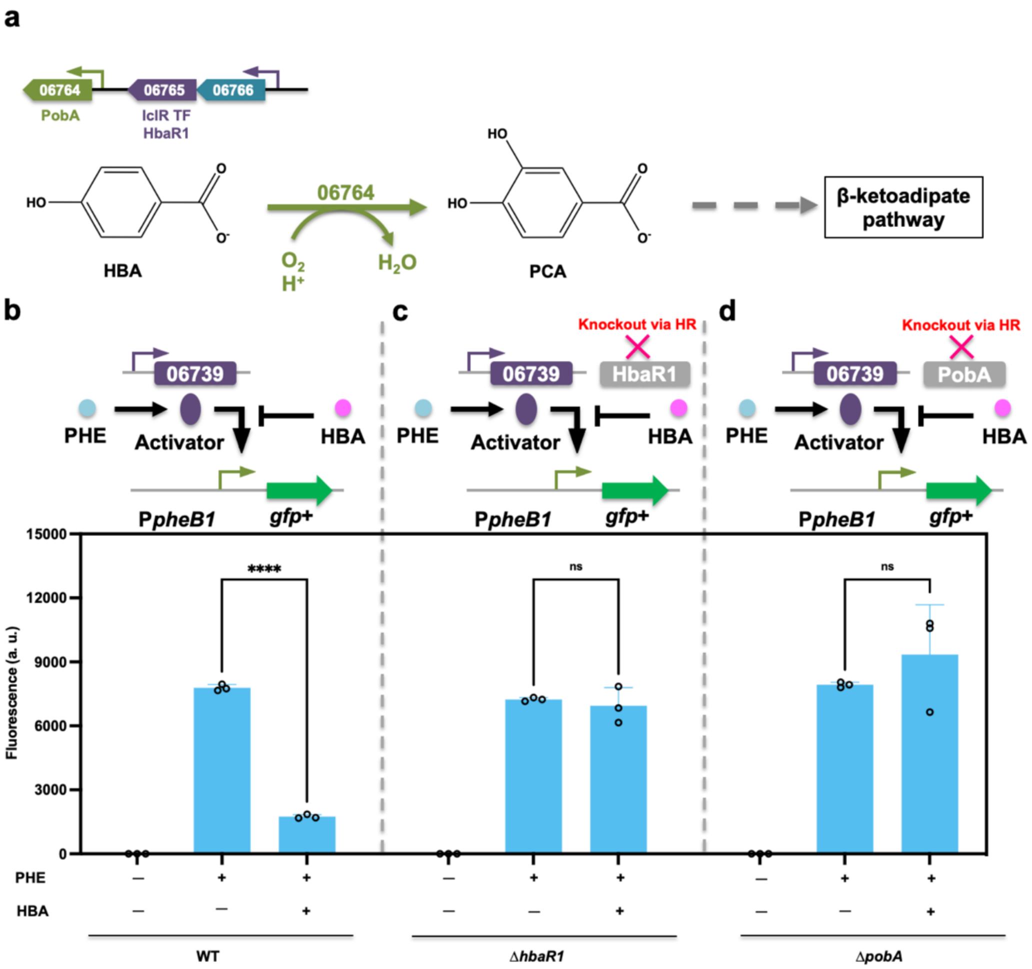

Supplementary Figure 10. Promoter activity of P*pheB1* in response to phenol and 4-hydroxybenzoate. a, Schematics depicting the funneling pathway for 4-hydroxybenzoate (HBA) and the annotated transcription factor. **b-d**, Proposed working models for promoter P*pheB1* during growth in minimal medium with phenol (PHE) and HBA (upper) and fluorescence of P*pheB1*-GFP+ in response to PHE and HBA in WT and knockout strain backgrounds (lower). The transcriptional construct P*pheB1*-GFP+ was expressed in the WT, Δ*hbaR1*, and Δ*pobA* strains. All cultures were grown with 1 g/L glucose and co-fed with either 0.3 g/L PHE or both PHE and 0.9 g/L HBA; fluorescence values were measured in early stationary phase and normalized to OD_{600}. Knocking out *hbaR1* and *pobA* reduced the inhibition of P*pheB1* by HBA in the presence of PHE, versus in PHE-only cultures (****, P <0.0001; ns, not significant, unpaired two-tailed *t*-test). All values represent the mean of triplicate cultures, with error bars depicting the standard deviation from that mean.

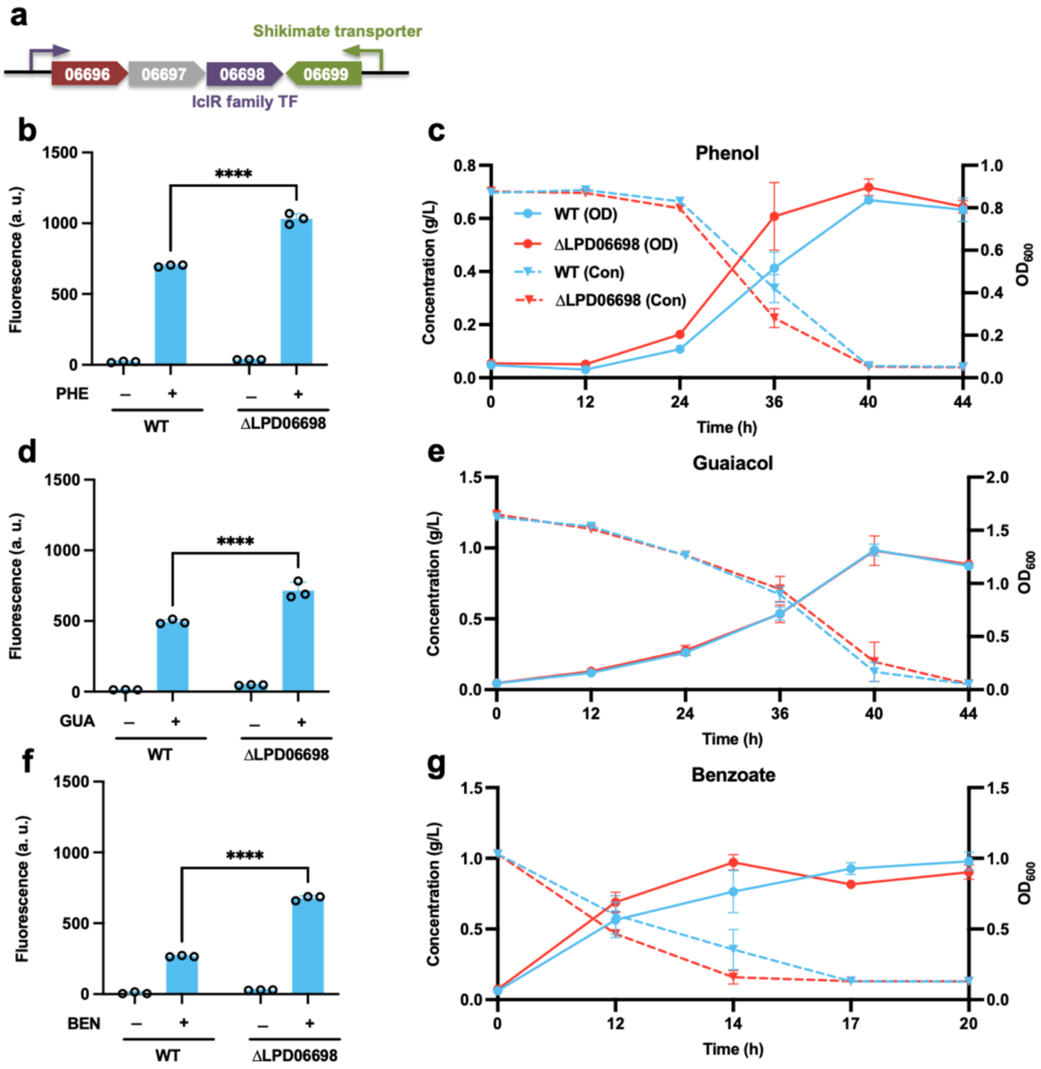

Supplementary Figure 11. Functional analysis of transcription factor LPD06698 in regulating shikimate transport relative to the catechol branch of the β-ketoadipate pathway. **a**, Schematic of the shikimate transport gene with an annotated transcription factor (TF); promoters are represented as small arrows and genes are shown with LPD gene numbers from the NCBI database (Refseq, CP003949.1). To test the regulatory effects of LPD06698, the upstream promoter region of LPD06699 was cloned in front of *gfp*+ and expressed in WT and the TF deletion mutant. **b**, **d**, **f**, The normalized fluorescence in the WT and ΔLPD06698 backgrounds was measured with and without supplemental phenol (PHE), guaiacol (GUA), or benzoate (BEN). In all cases, the ΔLPD06698 mutant exhibited significantly more fluorescence than the WT when the growth medium contained an aromatic carbon source (****, P <0.0001, unpaired two-tailed *t*-test). All fluorescence values were measured in the early stationary phase and normalized to OD_{600}. **c**, **e**, **g**, Comparison of cell growth (OD) and aromatic consumption (Con) of the WT and ΔLPD06698 strains. Cells were provided with 0.7 g/L phenol, 1.25 g/L guaiacol, or 1 g/L benzoate as the sole carbon source. All values represent the mean of triplicate cultures, with error bars depicting the standard deviation from that mean.

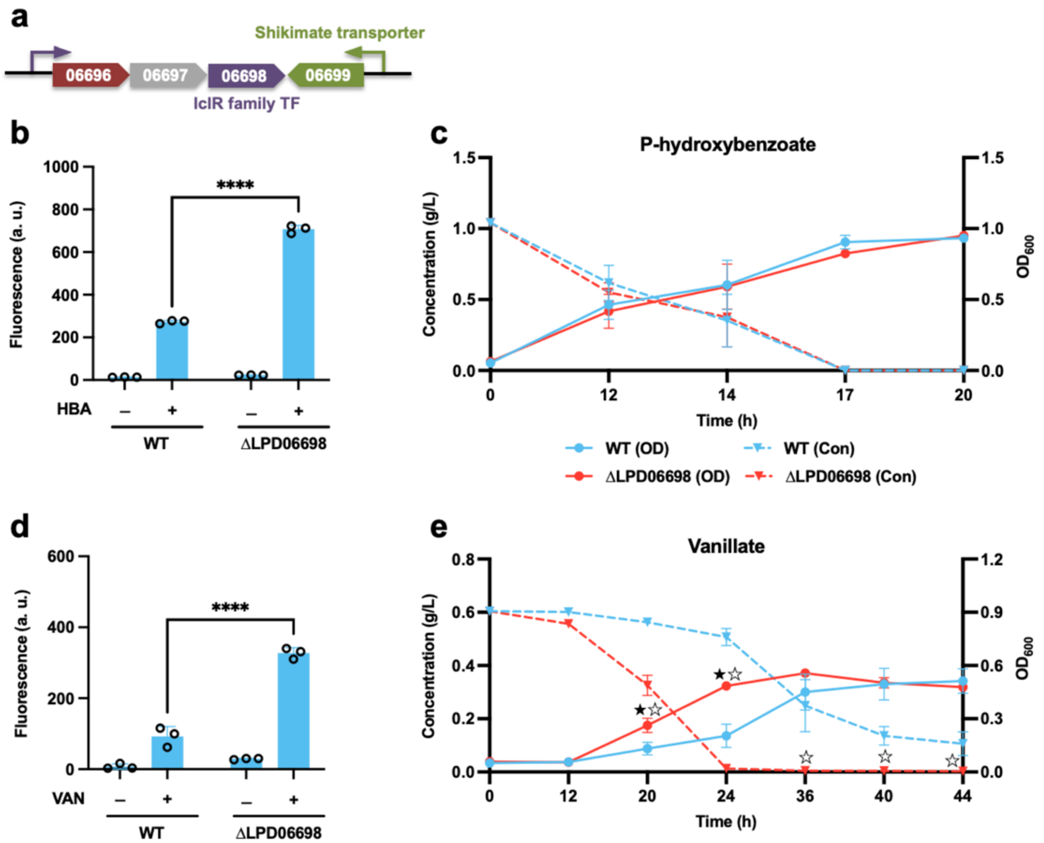

Supplementary Figure 12. Functional analysis of transcription factor LPD06698 in regulating shikimate transport relative to the protocatechuate branch of the β-ketoadipate pathway. a, Schematic of the shikimate transport gene with an annotated transcription factor (TF); promoters are represented as small arrows and genes are shown with LPD gene numbers from the NCBI database (Refseq, CP003949.1). To test the regulatory effects of LPD06698, the upstream promoter region of LPD06699 was cloned in front of *gfp+* and expressed in the WT and the TF deletion mutant strains. **b**, **d**, The normalized fluorescence in the WT and ΔLPD06698 backgrounds was measured with and without supplemental 4-hydroxybenzoate (HBA) or vanillate (VAN). In all cases, the ΔLPD06698 mutant exhibited significantly stronger fluorescence than the WT when the growth media contained an aromatic carbon source. All fluorescence values were measured in the early stationary phase and normalized to OD_{600} (****, P <0.0001, unpaired two-tailed *t*-test). **c**, **e**, Comparison of cell growth (OD) and aromatic consumption (Con) of the WT and ΔLPD06698 strains. Cells were provided with 1.0 g/L HBA or 0.6 g/L VAN as the sole carbon source. When grown on VAN, the behavior of the ΔLPD06698 mutant was significantly different from that of the WT (★, P_{OD} <0.05; ☆, P_{Con} <0.05; two-tailed mixed model ANOVA with Sidak's multiple comparisons). All values represent the mean of triplicate cultures, with error bars depicting the standard deviation from that mean.
