# Supplementary material for: Deciphering the transcriptional regulation of the catabolism of lignin-derived aromatics in Rhodococcus opacus PD630
Source: Commun Biol. 2022 Oct 19;5:1109. doi: 10.1038/s42003-022-04069-2 (PMC9582017; doi:10.1038/s42003-022-04069-2)
Supplement: Supplementary file 3 — Description of Additional Supplementary Files [file 42003_2022_4069_MOESM3_ESM.pdf]

Description of Additional Supplementary Files

File name: Supplementary Data 1

Description: Source data for the main figures

File name: Supplementary Data 2

Description: Source data for the supplementary figures
